# 

**Published:** 1871-06

**Authors:** 


### Books and Pamphlets Received.

A treatise on the Diseases of the Nervous System, By W. A. Hammond, M. D., with forty-five illustrations. New York, D. Appleton & Co., 1871. Breed, Lent & Co., Buffalo.

On the Physiological effects of Severe and Protracted Muscular Exercise; with special reference to its influence upon the excretisn of nitrogen. By Austin Flint Jr., M. D. New York, D. Appleton & Co., 1871.

The Eye in Health and Disease. Being a series of articles on the Anatomy and Physiology of the Human Eye, and its Surgical and Medical treatment. By B. Joy Jeffries, A. M., M. D. Boston, Alexander Moore, 1871. Breed, Lent & Co., Buffalo.

Students Chart of the Sympathetic Nerve. By Ralph M. Townsend, M. D. Philadelphia, Turner Hamilton.

Atlantic Monthly; The Nation; New York Observer; Little's Living Age; Peterson's Musical Monthly; American Educational Monthly; American Agriculturist: Phrenological Journal; Newspaper Reporter, &c.

The New York Observer year book and almanac, 1871. Sidney E. Morse, Jr. & Company, 37 Park Row, New York.

88th Annual Announcement of the Harvard University, Medical School. 1871-72. The year begins Sept. 28th and ends June 27th.

The "Fibrinous Crasis," Its cause a loss of albumen from the blood. By Rollin R. Gregg, M. D., Buffalo, N. Y.

The Detection of Criminal Abortion. By Ely Van De Warker, M. D., Syracuse, N. Y.

Seventeenth Annual Report of William J. Mullen, Prison Agent. Philadelphia, January 1871.

